# Interfacial Nucleation Pathways Governing Polymorph Selection Revealed by Synchrotron-Based *In Situ* GIWAXS and Molecular Simulations

**DOI:** 10.1021/jacs.6c06437

**Published:** 2026-07-07

**Authors:** Yu Liu, Xu Zhang, Xingfan Zhang, Mingdong Zhou, Xiaolong Li, Fumin Wang, Xubin Zhang, Alexey A. Sokol, You Lu, Thomas W. Keal, Weiwei Tang, C. Richard A. Catlow, Junbo Gong

**Affiliations:** † School of Chemical Engineering and Technology, 12605 Tianjin University, Tianjin 300072, People’s Republic of China; ‡ School of Chemical Engineering, 118412 Shenyang University of Chemical Technology, Shenyang 110142, People’s Republic of China; § Kathleen Lonsdale Materials Chemistry, Department of Chemistry, 4919 University College London, London WC1H 0AJ, U.K.; ∥ Chinese Academy of Sciences, Shanghai Advanced Research Institute, Shanghai Synchrotron Radiation Facility, Shanghai 201204, People’s Republic of China; ⊥ School of Chemistry, Cardiff University, Park Place, Cardiff CF10 3AT, U.K.; # STFC Scientific Computing, Daresbury Laboratory, Warrington WA4 4AD, U.K.

## Abstract

Gas–liquid interfacial nucleation can influence organic crystallization, yet its mechanistic role in polymorph selection remains poorly understood. Here, we demonstrate that nucleation at the gas–liquid interface of flufenamic acid solutions governs polymorph selection and gives rise to a pronounced concentration-dependent polymorphism, wherein low initial concentrations favor the nucleation of form I, higher concentrations yield form III, and intermediate concentrations selectively produce the metastable form IV. *In situ* synchrotron-based grazing-incidence wide-angle X-ray scattering (GIWAXS) directly resolves the formation and evolution of prenucleation assemblies at the interface, revealing three distinct interfacial molecular evolution pathways correlated with the emergence of specific polymorphs. Molecular dynamics (MD) simulations combined with energy calculations within the hybrid quantum mechanics/molecular mechanics (QM/MM) embedded-cluster framework further reveal interfacial enrichment, orientational bias, and conformer-dependent stabilization of stacking motifs, providing a microscopic interpretation of the experimentally observed selectivity. Together, these results establish the gas–liquid interface as an active structural selector that reshapes molecular organization prior to nucleation, offering a mechanistic framework for understanding and controlling polymorphism in evaporation-driven crystallization.

## Graphical abstract

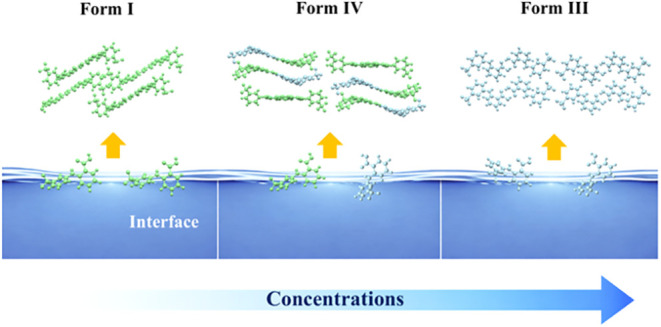

## 1 Introduction

Polymorphism is a widely observed phenomenon in organic crystallization and plays a decisive role in determining the physicochemical properties of molecular solids, including their solubility, stability, and bioavailability.^1^ Despite extensive experimental and theoretical efforts, controlling polymorph formation remains a central challenge, largely because polymorph outcomes are often governed by subtle structural and kinetic factors operating during the earliest stages of nucleation.^2–5^ In many crystallization protocols, particularly those involving solvent evaporation, polymorph outcomes are highly sensitive to experimental details and frequently lack reproducibility, suggesting an incomplete mechanistic understanding of nucleation at the molecular level.^6^

Evaporation-driven crystallization inherently involves the presence of a gas–liquid interface, where solvent removal, concentration gradients, and anisotropic molecular environments coexist.^7–11^ A growing body of experimental and theoretical work has suggested that such interfaces can influence nucleation behavior.^12–15^ For example, Yu et al.^16^ reported the gas interface-induced polymorph selection of D-arabitol and interpreted the phenomenon by the different structure of the liquid surface through molecular dynamics (MD) simulations. Lu et al.^17,18^ revealed the important roles of water arrangement at the air–water interface during the nucleation of ibuprofen through MD simulations and surface-specific vibrational sum-frequency generation (SFG) spectroscopy. However, despite these advances, most existing studies rely on indirect evidence, such as nucleation statistics, induction time analysis, or postcrystallization characterization, and do not directly resolve the molecular organization at the interface preceding nucleation. Consequently, whether and how interfacial molecular assembly directs polymorph selection remains unresolved, particularly for conformationally flexible molecules capable of adopting multiple packing motifs.

A major obstacle in addressing this question lies in the lack of experimental techniques capable of directly probing molecular structure and its evolution at the gas–liquid interface. Bulk-averaged spectroscopic methods, such as NMR,^19–21^ IR,^22,23^ and Raman spectroscopy,^24,25^ provide valuable insights into solution-state association and conformational equilibria but are insensitive to interfacial ordering and orientational anisotropy. Conversely, *ex situ* diffraction techniques capture only the final crystalline products and cannot access the transient molecular assemblies that precede nucleation. Consequently, direct experimental evidence linking interfacial molecular organization to polymorph outcomes has remained scarce, despite longstanding recognition of the importance of interfaces in crystallization phenomena.

*In situ* grazing-incidence wide-angle X-ray scattering (GIWAXS) offers a unique opportunity to overcome these limitations by selectively probing near-surface structural correlations with high temporal resolution.^17,26–28^ While GIWAXS has been widely applied to study thin-film formation and crystallization in inorganic and hybrid systems, its application to resolving interfacial nucleation mechanisms of small organic molecules during evaporation remains largely unexplored. When combined with molecular simulations, such an approach enables us to build a connection between the experimentally observed interfacial information and the underlying molecular interactions, orientational preferences, and conformational energetics that shape the nucleation pathways.

Here, we investigate the evaporation-driven crystallization of flufenamic acid (FFA), a conformationally flexible pharmaceutical molecule exhibiting rich polymorphism,^29,30^ as a model system to elucidate the role of gas–liquid interfacial nucleation. By systematically varying concentration and crystallization conditions, we uncover a pronounced concentration-dependent polymorphism, characterized by distinct polymorphic outcomes at low, intermediate, and high concentrations, that has not been previously resolved in evaporation-driven crystallization of organic molecules and cannot be rationalized by bulk solution behavior alone. Using synchrotron-based *in situ* GIWAXS, we directly visualize the formation and evolution of prenucleation assemblies at the gas–liquid interface in real time and identify distinct interfacial molecular evolution pathways leading to different polymorphic forms.^31^ Molecular dynamics simulations combined with multiscale quantum mechanics/molecular mechanics (QM/MM) calculations provide a microscopic interpretation of interfacial enrichment, orientational bias, and conformer-dependent stabilization of stacking motifs. Together, these results establish the gas–liquid interface as an active structural selector in evaporation-driven crystallization and offer new mechanistic insight into polymorph control in organic molecular solids.

## 2 Results and Discussion

### 2.1 Crystal Structure Analysis

The molecular conformations of flufenamic acid (FFA) and the crystal structures of forms I, III, and IV were systematically analyzed, with the crystallographic parameters summarized in Tables S1 and S2 and representative packing motifs shown in Figure 1. The reported basic physical properties of these polymorphs, including melting points, relative thermodynamic stability, transition temperature, and available temperature-dependent solubility data, are summarized in Table S3.

**Figure 1:**
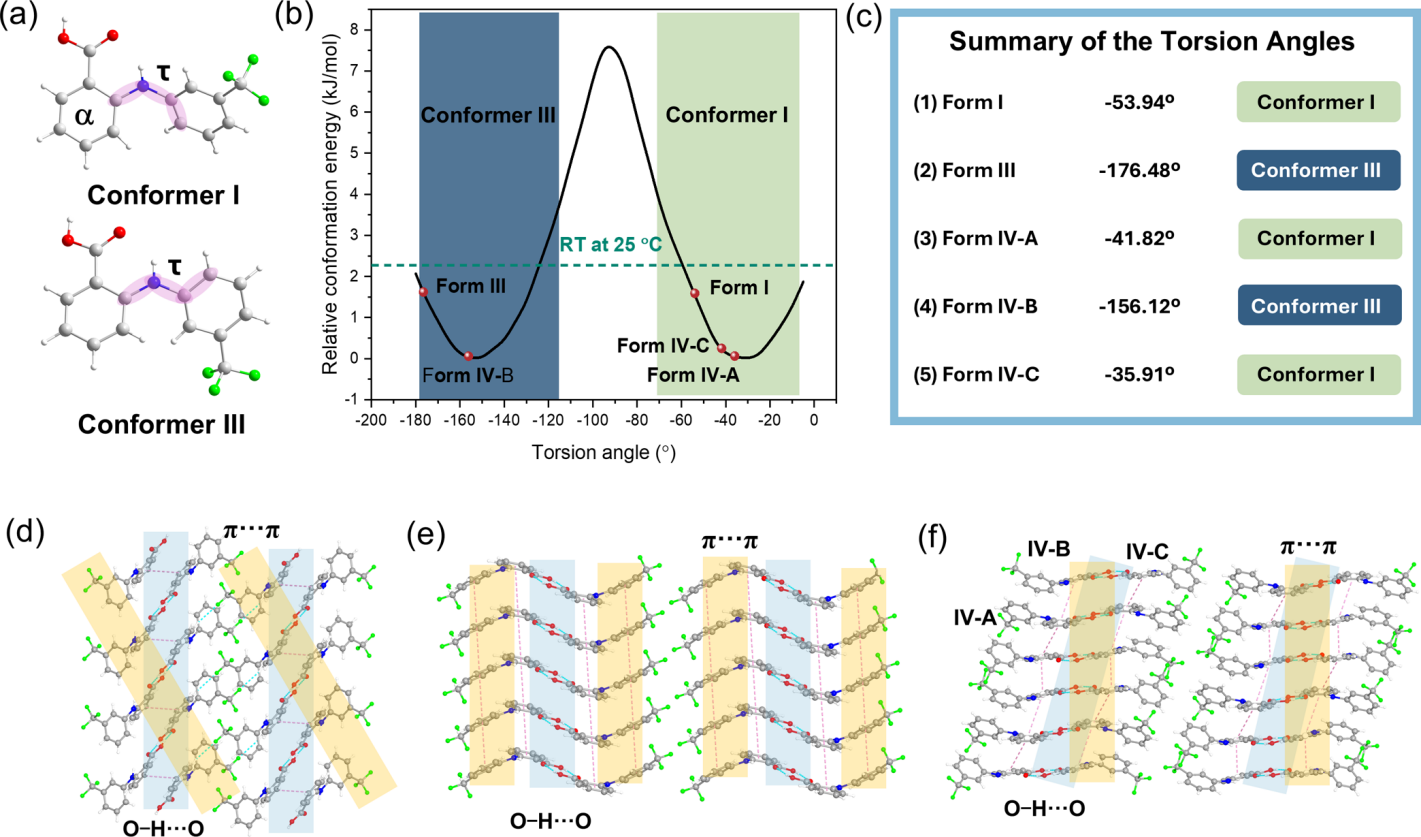
Crystal packing features of FFA polymorphs. (a) Molecular structures of representative conformer I and conformer III as observed in FFA forms I and III, respectively. (b) Calculated conformational energy profile of FFA as a function of the torsion angle. (c) Comparison of key torsion angles for FFA molecules in different crystal structures. (d–f) Crystal packing motifs of FFA form I (d), form III (e), and form IV (f), highlighting their common supramolecular packing features. Atom colors: C, gray; H, white; O, red; N, blue; and F, green.

Despite their polymorphic diversity, all three forms share a common supramolecular framework: FFA molecules first assemble into carboxylic acid hydrogen-bonded dimers (O–H···O), which act as primary synthons and further expand through π···π stacking interactions to generate columnar packing motifs. Intercolumn interactions are relatively weak and are mainly mediated by contacts involving the −CF_3_ groups. The key structural variations arise from differences in molecular conformation, which directly modulate the stacking efficiency and packing density, as revealed in Table S1.

As illustrated in Figure 1a, FFA exhibits two representative conformations that differ primarily in the torsion angle (τ) between the two aromatic rings. A potential energy scan (PES) along τ (Figure 1b) reveals two distinct low-energy minima corresponding to a twisted conformer (conformer I) and a more planar conformer (conformer III), separated by a moderate energy barrier of approximately 7.65 kJ mol^–1^. This relatively low barrier indicates that interconversion between the two conformers is facile under ambient conditions, allowing both conformations to be accessible during crystallization.

These conformational differences lead to distinct π···π stacking geometries (Figure 1d–f). The twisted conformer I favors slipped stacking with reduced packing efficiency, as observed in form I, whereas the planar conformer III enables denser and more compact stacking columns characteristic of form III. Form IV exhibits multiple stacking arrangements arising from the coexistence of both conformers, resulting in an overall packing density intermediate between those of forms I and III.

Overall, the crystal structure analysis demonstrates that FFA polymorphism is fundamentally governed by conformer-dependent π···π stacking. This conformational stability provides a structural basis for the concentration- and interface-dependent polymorph selection observed during crystallization, as explored in the following sections.

### 2.2 Crystallization Results

To investigate the effects of interfacial nucleation on the FFA polymorph, four typical crystallization experiments were carried out: evaporation of a thin liquid film, static evaporation of the bulk solution, evaporation of the bulk solution with agitation, and evaporation with the addition of the second component to destroy the surface, as illustrated in Figure 2a. The polymorph outcomes with different initial concentrations for the four crystallization methods are shown in Tables S4–S7 and Figure 2.

**Figure 2:**
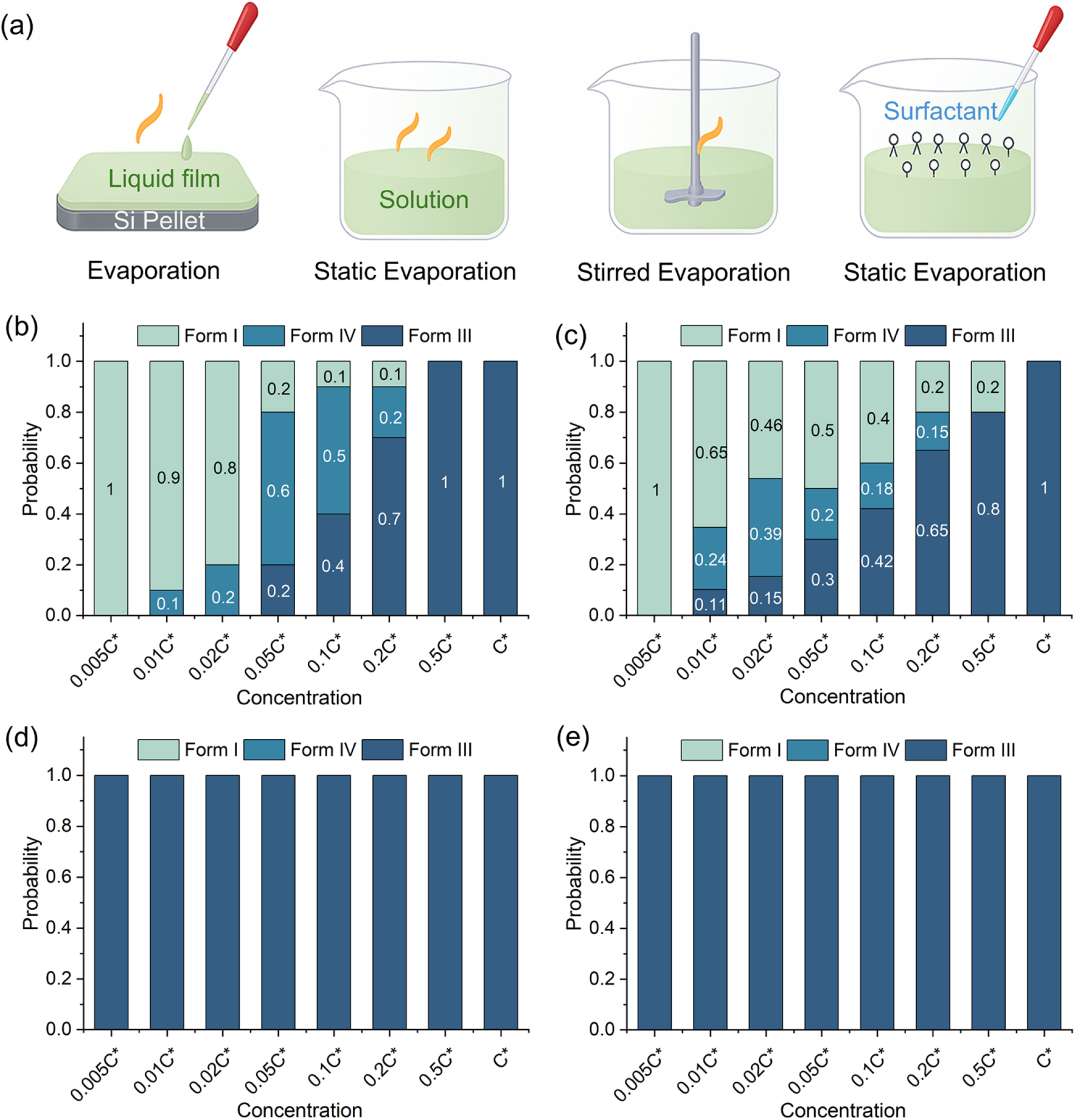
Crystallization outcomes showing the concentration-dependent polymorph selection at the gas–liquid interface. (a) Schematic illustration of the experimental setup: evaporation of the liquid film for surface nucleation, stirred evaporation of the bulk solution for bulk nucleation, static evaporation of the bulk solution, and static evaporation with adding surfactant. (b–e) Polymorphic outcomes of FFA from methanol with different initial concentrations in four crystallization methods: evaporation of liquid film (b), static evaporation of bulk solution (c), stirred evaporation of bulk solution (d), and evaporation with surfactant (e). C* represents the saturated concentration of FFA-methanol solution at 298.15 K.

We first examined crystallization occurring strictly at the quiescent surface (Figure 2b). Here, C* represents the saturated concentration of FFA in methanol at 298.15 K (*i.e.*, C* = 0.3445 g mL^–1^). The resultant polymorphs displayed a clear and reproducible dependence on the initial concentration. At low initial concentrations (*e.g*., 0.005C*), only metastable form I crystallized, while only stable form III nucleated at saturated initial concentrations. Within intermediate concentrations, the nucleation of FFA exhibited stochastic behavior. Repeated experiments performed under the same controlled experimental conditions could yield different polymorphic outcomes, including form I, form III, form IV, and occasionally mixed forms I/III. The polymorph probability was therefore calculated as the occurrence frequency of each form across repeated independent experiments. As the initial concentration increased, form III displayed an increasing tendency to nucleate from methanol compared to the other forms. In general, the nucleation of FFA at the gas–liquid interface exhibits strong concentration effects, *i.e*., a high initial concentration promoted the nucleation of form III, whereas a low initial concentration favored the nucleation of form I, and a moderate concentration occasionally yielded metastable form IV.

We then investigated static slow-evaporation crystallization of bulk solution (Figure 2c), which is commonly used for single crystal preparations, to test whether the interfacial effect can indeed influence the actual laboratory crystallization. Remarkably, static slow evaporation showed a broadly similar concentration-dependent tendency to the interfacial experiments: low concentrations yielded form I, high concentrations yielded form III, and form IV emerged only within the narrow intermediate region. It is noteworthy that the uncommon metastable form IV appeared within the concentrations ranging from 0.01C* to 0.2C*, which encompasses the concentration ranges reported by López-Mejías et al.^29^ for preparing form IV through the slow evaporation method. Because slow evaporation inherently generates a stable gas–liquid interface and steep surface concentration gradients, these results indicate that interfacial nucleation persists throughout the entire evaporation process and thus influences the macroscopic crystallization outcomes used in practical workflows. However, possible contributions from bulk nucleation during static slow evaporation, particularly at high concentrations or late evaporation stages, cannot be completely excluded. This may partly explain why the polymorphic distributions observed in the static slow-evaporation experiments do not exactly reproduce those obtained in the surface nucleation experiments. We also monitored mass loss under static evaporation conditions to examine variations in evaporation rate (Figure S5). Because evaporation proceeded slowly, only minor differences in evaporation rate were observed among solutions with different initial concentrations. Therefore, macroscopic differences in evaporation rate are less likely to be the primary cause of the distinct polymorphic outcomes.

In comparison, we carried out parallel crystallization experiments in a bulk solution with strong stirring to suppress surface nucleation (Figure 2d). Under these fully mixed conditions, the concentration dependence vanished completely, and form III was obtained across the entire studied concentration range. This contrast shows that the bulk solution lacks the structural bias required to differentiate polymorph outcomes, confirming that the concentration-dependent behavior is not a bulk thermodynamic effect but instead arises from interfacial nucleation.

Finally, to verify further that the interface is the mechanistic origin of this polymorphic behavior (Figure 2e), we intentionally disrupted the surface layer by adding a nonionic surfactant (Tween 80, Figure S3). This strategy suppressed interfacial nucleation successfully and produced exclusively form III across all concentrations. Solubility measurements indicated that Tween 80 had a negligible influence on FFA dissolution (Table S8), with mole-fraction solubilities of 0.0458 in pure methanol and 0.0456 in Tween-80-containing methanol. No additional solid phase or new polymorph was detected after Tween 80 addition. The enrichment of surfactant at the interface reduces the local concentration of FFA molecules and thus inhibits the self-assembly of FFA at the interface and forces crystallization to proceed along the bulk-like nucleation pathway, confirming the causal role of the gas–liquid interface.

Together, these experiments demonstrate that the evaporation of FFA exhibits an interesting concentration-dependent polymorphism that arises from interfacial nucleation pathways. When the interface is preserved (static surface or slow evaporation), this pathway remains active and yields multiple polymorphs. When the interface is disrupted (stirring or surfactant), the system collapses to the bulk pathway and produces Form III alone.

### 2.3 Solution States in Bulk Solution

To identify the molecular states present prior to nucleation, we probed FFA in bulk methanol over a wide concentration range using ^1^H NMR spectroscopy (Figure 3a–c). The spectra show concentration-dependent chemical shift changes in the aromatic region. In particular, protons on the α-ring (*e.g*., H_4_ and H_5_) display pronounced upfield shifts with increasing concentration (Figure 3b), consistent with enhanced aromatic shielding effects by π···π stacking. Although partial overlap prevents full resolution of H_12_ and H_13_ on the β-ring (Figure 3a), their net upfield displacement further supports the progressive strengthening of aromatic interactions with concentration.

**Figure 3:**
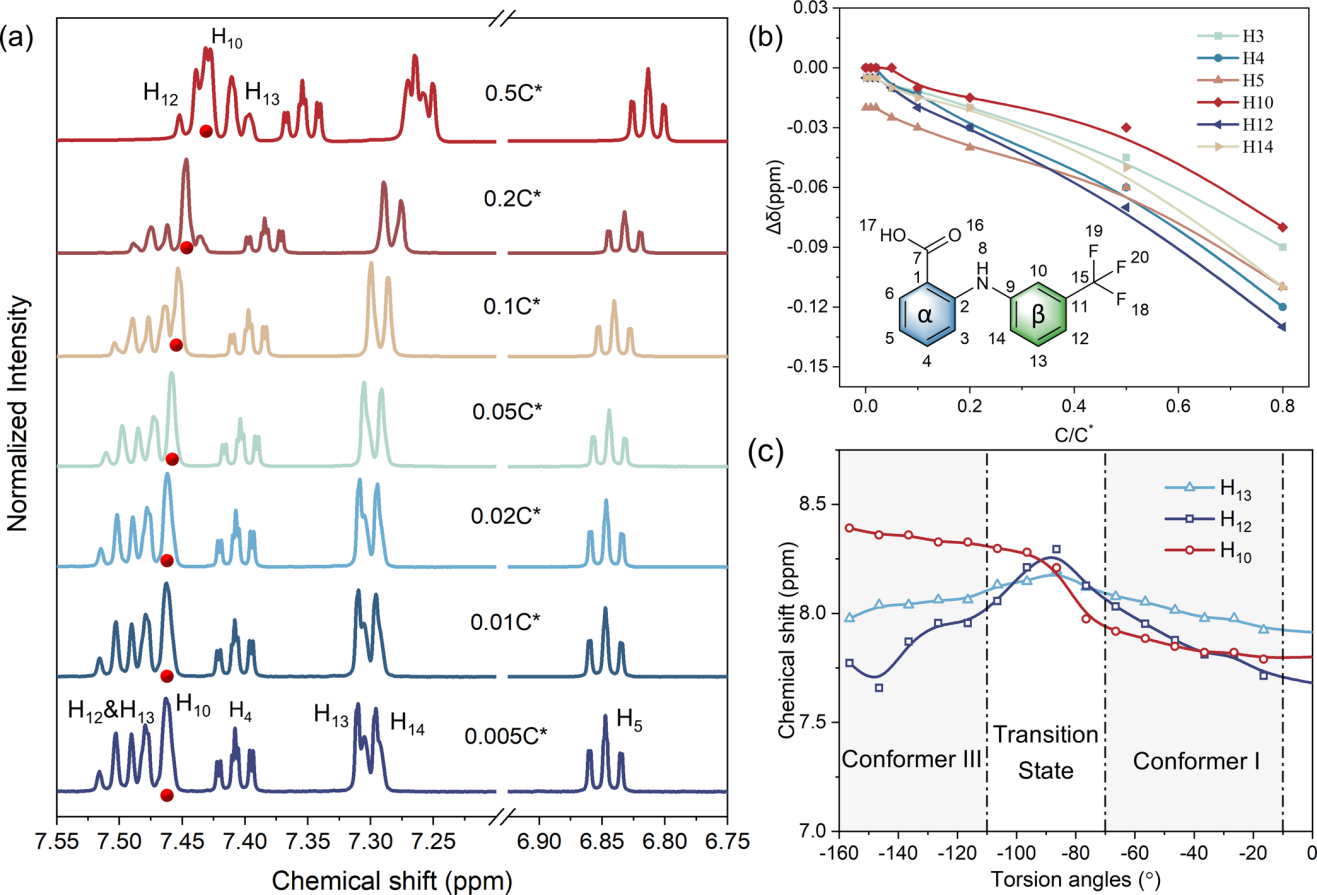
Concentration-dependent NMR signatures and conformational assignment of FFA in bulk solution. (a) ^1^H NMR spectrum of FFA in methanol with different concentrations at 25 °C, and the red sphere represents the position of H_10_. The chemical shift of H_12_ and H_13_ partially overlaps and thus is difficult to separate completely. (b) ^1^H NMR chemical shift changes of FFA as a function of concentration. (c) Calculated changes in chemical shift as a function of torsion angle τ for three selected hydrogen atoms (H_10_, H_12_, H_13_), which exhibit the most significant changes in chemical shift when conformation changes.

Beyond uniform shielding effects, a notable feature is the reversal of the relative peak ordering among H_10_, H_12_, and H_13_ as concentration increases (Figure 3a). Because these protons are highly sensitive to the torsion between the two phenyl rings, such peak reordering indicates a concentration-dependent redistribution of conformer populations rather than a simple bulk dielectric effect. To identify this assignment, we computed chemical shifts for H_10_, H_12_, and H_13_ along a torsion-angle scan (Figure 3c). The calculations reproduce the experimentally observed trend. In conformer III, δ­(H_10_) lies between δ­(H_12_) and δ­(H_13_), whereas in the more twisted basin (Conformer I), H_10_ becomes the most upfield signal. A comparison of experiment and computation therefore indicates that bulk methanol solutions are conformationally adaptive, *i.e*., conformer III becomes increasingly populated at higher concentrations, while conformer I is favored at lower concentrations.

Taken together, the NMR results demonstrate that FFA in bulk methanol undergoes concentration-dependent changes in aromatic association and conformational equilibrium. However, these bulk-averaged signatures do not, by themselves, imply the presence of polymorph-directing ordered clusters. Instead, they provide the initial solution state that can be selectively amplified under bulk conditions. The structural organization and selection occurring at the gas–liquid interface, however, cannot be resolved by NMR and are instead elucidated by the subsequent *in situ* GIWAXS measurements.

### 2.4 Cluster Evolution at the Gas–Liquid Interface

*In situ* synchrotron-based GIWAXS was employed to directly track the molecular organization preceding nucleation at the gas–liquid interface. The experimental procedure and the representative scattering features are summarized in Figures 4a,b and S6. Time-resolved 2D patterns for three characteristic concentrations (0.005C*, 0.2C*, and 0.9C*) are compiled in Figure 4c–e, and other concentrations exhibiting similar trends are given in Figure S7. Integrated profiles *I*(*q*) (Figure 4f–h) were further used to resolve subtle changes in correlation distances during the prenucleation stage. To clarify the structural basis for assigning these diffuse features, the observed q values were converted into real-space correlation lengths using *d* = 2π/q and compared with representative intermolecular distances extracted from FFA crystal structures and MD-derived assemblies (Table S9), with the corresponding assignments discussed below.

**Figure 4:**
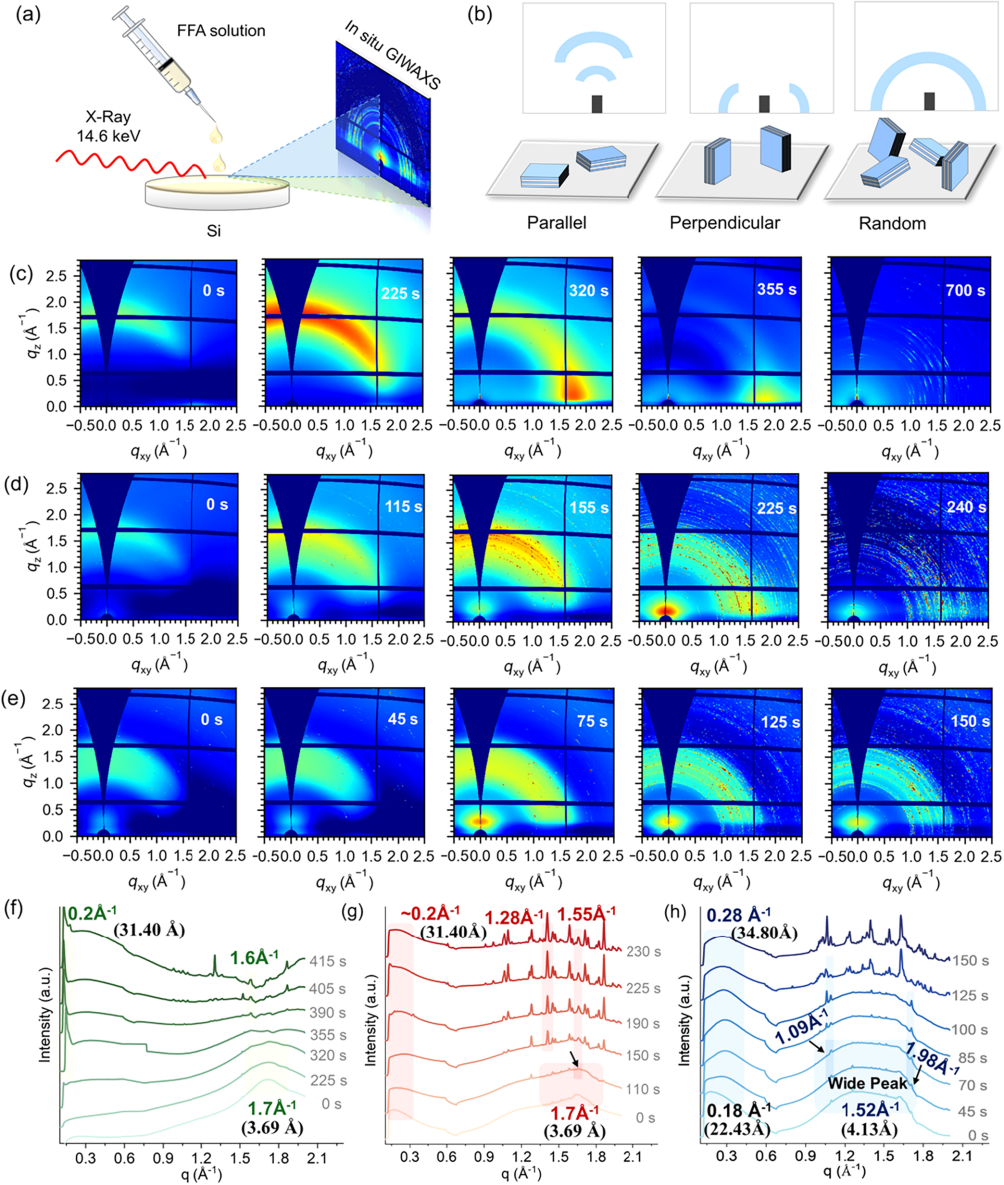
*In situ* GIWAXS measurements of the FFA surface nucleation under different concentrations. (a) Illustration of the synchrotron-based *in situ* GIWAXS setup for surface nucleation of FFA. (b) Typical scattering patterns associated with different packing orientations. (c–e) *In situ* GIWAXS maps of FFA solutions at (c) 0.005C*, (d) 0.2C*, and (e) 0.9C*. (f–h) Time-resolved GIWAXS integrated I­(q) profiles at 0.005C* (f), 0.2C* (g), and 0.9C* (h).

At low concentration (0.005C*), the original scattering pattern shows a weak, elongated arc centered at q≈1.7 Å^–1^ (Figure 4c), corresponding to a dominant short-range correlation distance of 3.69 Å (*d* = 2π/q). Such a length scale is consistent with stacking-related contacts in aromatic systems (Figures S8 and S9) and indicates the presence of short-range aromatic correlations in the solution during evaporation. However, the diffuse nature of the integrated peak, together with the absence of peak sharpening, indicates that these correlations do not correspond to well-defined stacking motifs. Instead, the signal reflects a transient and dynamic π···π association at the initial stage. Notably, the corresponding two-dimensional patterns reveal a pronounced evolution in azimuthal intensity distribution. At the initial stage, the scattering arc is nearly parallel, suggesting that these interfacial motifs exhibit a degree of preferred orientation relative to the surface. With evaporation, a transient enhancement of scattering intensity perpendicular to the interface emerges, reflecting a reorientation of interfacial clusters as local concentration and intermolecular coupling increase. At later stages, this orientational anisotropy relaxes, and the azimuthal intensity becomes more uniformly distributed. An additional low-q feature emerges around *q* ≈ 0.2 Å^–1^ (*d* ≈ 31.4 Å), indicative of mesoscale density correlations or interaggregate ordering within the near-surface region. Ultimately, the diffuse features are replaced by sharp Bragg reflections (Figure 4c, late stage; Figure 4f), marking the onset of crystalline order and the formation of form I.

At intermediate concentrations yielding form IV (0.2C*), the interfacial layer exhibits a different behavior. From the earliest stages, both a wide-angle arc near *q* ≈ 1.7 Å^–1^ and a low-q signal are simultaneously present (Figure 4d), implying the coexistence of short-range stacking correlations and mesoscale ordering already at the earliest stage. As evaporation proceeds, the initially broad stacking-related feature progressively resolves into multiple emerging peaks in the *q* ∼ 1.2–1.6 Å^–1^ region. This evolution indicates a transition from a unimodal local packing correlation to a multimodal set of characteristic distances, consistent with the development of more structurally diverse, yet increasingly ordered, interfacial assemblies. Unlike the low-concentration regime, the azimuthal intensity distributions evolve from nearly parallel to anisotropic within the evaporation process, with no emergence of the transition state (perpendicular orientations). The subsequent appearance and growth of sharp Bragg reflections signify crystallization, correlating with the formation of form IV, which accommodates mixed conformations and diverse stacking geometries in the crystal lattice.

At 0.9C*, the interfacial assembly pathway is further accelerated. The early-stage scattering patterns are dominated by a pronounced low-q signal at *q* ≈ 0.18 Å^–1^ (*d* ≈ 34.9 Å) and a broad arc centered at *q* ≈ 1.4 Å^–1^ (*d* ≈ 4.48 Å). Compared with lower concentrations, the shift of the wide-angle diffuse maximum from ∼1.7 to ∼1.4 Å^–1^ suggests a distinct dominant local packing correlation at the interface, consistent with a change in the prevalent stacking geometry or conformer composition in the interface. Importantly, the azimuthal intensity distribution of the wide arc is more dispersive and does not change a lot among the evaporation, indicating no preferred orientations of the initial assembly. As evaporation proceeds, the low-q feature gradually shifts toward higher q and stabilizes at around *q* ≈ 0.28 Å^–1^, implying a reduction in the characteristic mesoscale correlation length during evaporation, which may reflect progressive densification or reorganization of interfacial aggregates under increasing supersaturation. Weak higher-q features persist throughout the process, indicating the presence of short-lived ordered domains within the interfacial layer prior to the emergence of definitive Bragg peaks. The final stage is marked by the growth of sharp reflections (Figure 4e,4h), consistent with the formation of form III crystals at high concentration.

These *in situ* GIWAXS observations thus provide direct experimental evidence that dilute solutions evolve from dynamic stacking-related local correlations in parallel orientations to anisotropic, mesoscale ordering before crystallizing as form I. Intermediate solutions develop multimodal local correlations consistent with mixed packing requirements of form IV, and concentrated solutions exhibit a distinct stacking-related correlation and faster reorganization culminating in form III. These observations provide direct experimental evidence that the gas–liquid interface can act as an active structural selector that reshapes prenucleation order and thereby directs polymorph outcomes.

### 2.5 Modeling Results

Classical molecular dynamics (MD) simulations were performed to probe the early-stage structural evolution of FFA during evaporation in bulk solution and at the gas–liquid interface at different initial concentrations. The simulation reveals clear interfacial effects on molecular distribution, orientation, and interaction patterns that are absent in bulk solution (Figure 5).

**Figure 5:**
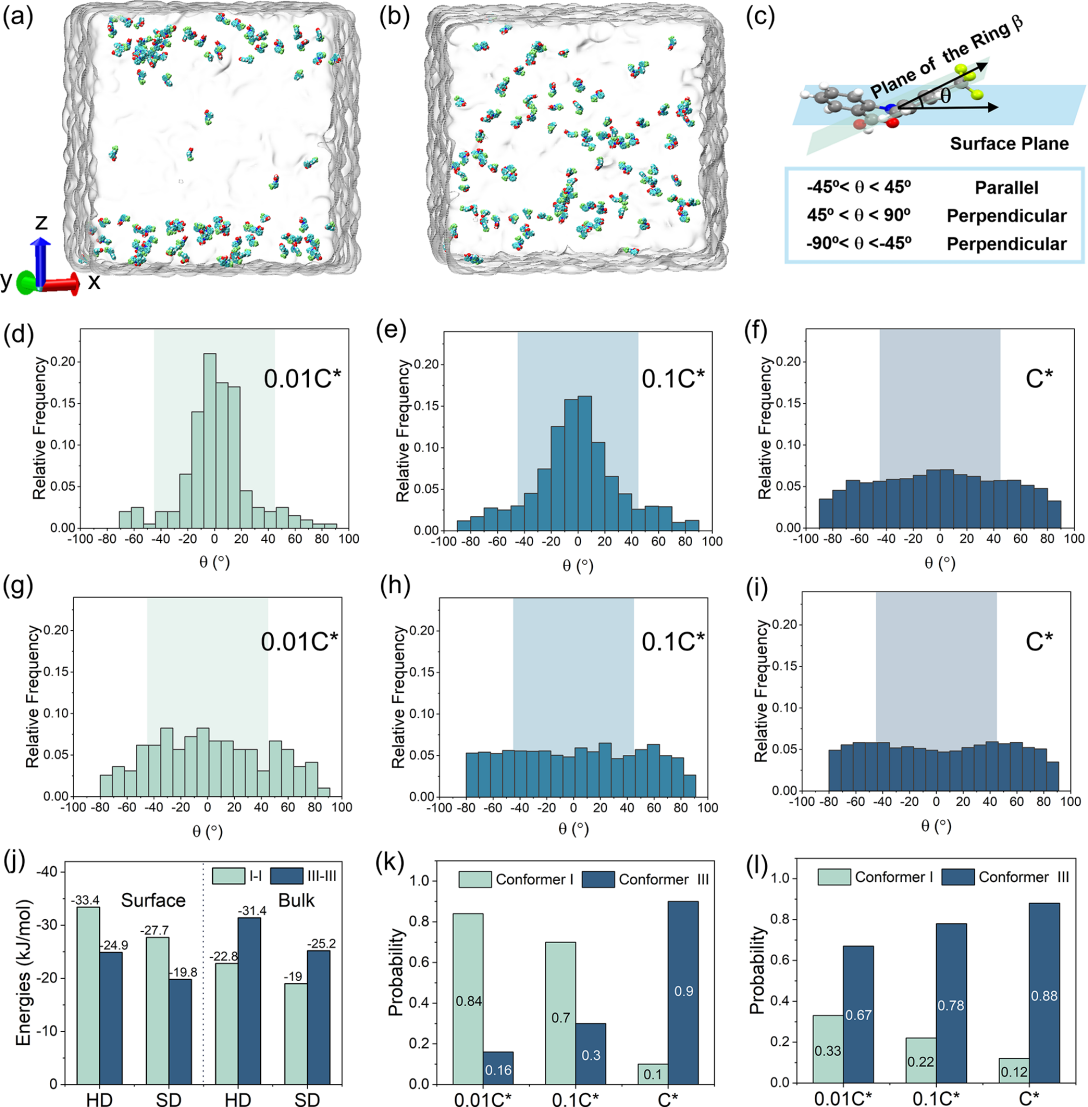
Molecular simulations of FFA assemblies at the gas–liquid interface and in bulk solution. (a, b) Representative MD snapshots illustrating the spatial distribution of FFA molecules at the gas–liquid interface (a) and in bulk solution (b) at 0.1C*. Methanol molecules are rendered in white for clarity. (c) Definition of the molecular orientation angle θ between the aromatic ring plane and the surface plane. (d–f) Probability distributions of θ for FFA molecules at the gas–liquid interface at (d) 0.01C*, (e) 0.1C*, and (f) C*. (g–i) Probability distributions of θ for FFA molecules in bulk solution at (g) 0.01C*, (h) 0.1C*, and (i) C*. (j) Averaged interaction energies of FFA dimers at the gas–liquid interface and in bulk solution, including hydrogen-bonded dimers (HD) and stacked dimers (SD). (k, l) Conformer distributions of FFA at the gas–liquid interface (k) and in bulk solution (l) at the corresponding concentrations. All statistical analyses are based on 20 selected snapshots.

Representative MD snapshots (Figures 5a,b and S10) show that FFA molecules remain homogeneously distributed in bulk solution, whereas pronounced interfacial enrichment occurs at the gas–liquid interface. This enrichment becomes more significant with increasing concentration, leading to the formation of a dense near-surface molecular layer (Figure S10). Local density profiles were further extracted along the surface-normal direction to distinguish the nominal initial bulk concentration from the local interfacial composition (Figure S11). For each initial concentration, the interfacial system shows a higher FFA/MeOH ratio near the gas–liquid interface than the corresponding bulk system, confirming preferential FFA accumulation at the interface. This analysis provides a semiquantitative basis for the subsequent discussion of interfacial orientation, conformer redistribution, and association motifs.

A key distinction between bulk and interfacial environments lies in molecular orientation, as shown by the comparison between the interfacial distributions in Figure 5d–f and the bulk distributions in Figure 5g–i. At the gas–liquid interface, the orientation distributions of FFA molecules are strongly biased and evolve with concentration (Figure 5d–f). At low concentration, molecular orientations are predominantly parallel to the interface, consistent with the anisotropic constraints imposed by the surface and with the GIWAXS results (Figure 4). At intermediate concentration, the distribution broadens and additional orientational states emerge, whereas at high concentration it becomes more dispersed, consistent with increased molecular crowding and reduced orientational selectivity. In contrast, FFA molecules in bulk solution exhibit nearly isotropic orientation distributions across all concentrations (Figure 5g–i), confirming the absence of such interfacial orientational bias. To quantify this orientational bias, we defined the parallel-orientation fraction, F_45_, as the percentage of FFA molecules with |θ| ≤ 45°, where θ = 0° corresponds to the aromatic ring plane being parallel to the gas–liquid interface. The value decreases from approximately 88.5% at 0.01C* to 81% at 0.1C* and further to 50% at C*, confirming the concentration-dependent weakening of surface-induced orientational selectivity.

The interfacial orientational bias can be understood in terms of interfacial confinement and anisotropic solvation. Unlike in the bulk solution, where FFA molecules are surrounded by a more isotropic methanol solvation shell, the gas–liquid interface exhibits a surface-normal gradient in local composition and solvation. These gradients couple with the anisotropic chemical structure of FFA, which contains polar hydrogen-bonding groups as well as hydrophobic aromatic and CF_3_-containing moieties (Figure 1a). Consequently, at low concentrations where FFA–FFA interactions are relatively weak, the interfacial solvation field favors specific molecular orientations. As concentration increases, intermolecular π-stacking, hydrogen bonding, and steric crowding increasingly compete with this surface-induced orientational preference, leading to broader orientation distributions. Thus, the concentration effect is better understood as a reweighting of conformer-dependent hydrogen-bonded and π-stacked association motifs.

This reweighting is also reflected in the interaction energies of representative FFA dimers (Figure 5j), calculated using the QM/MM approach. At the gas–liquid interface, the interaction energies of I–I dimers are −33.4 and −27.7 kJ mol^–1^ for hydrogen-bonded dimers (HD) and π···π stacked dimers (SD), respectively, which are more negative than those of III–III dimers (−24.9 and −19.8 kJ mol^–1^). This indicates that conformer-I-based association motifs are energetically preferred at the interface. In contrast, in bulk solution, III–III dimers become more stable, with interaction energies of −31.4 and −25.2 kJ mol^–1^ for HD and SD, respectively, which are more negative than those of the corresponding I–I dimers in Figure 5j. This reversal demonstrates that the gas–liquid interface reorders the relative energetic preference of conformer-dependent association motifs.

Conformer population analysis (Figure 5k,l) further highlights the distinction between bulk and interfacial conformational behavior. In bulk solution, conformer III remains dominant across the concentration range, accounting for approximately 67%, 78%, and 88% of the FFA population at 0.01C*, 0.1C*, and C*, respectively. This trend is consistent with the NMR observations, which indicate enhanced aromatic stacking and conformational redistribution toward conformer III upon concentration increase. In contrast, the interface shows a pronounced enrichment of conformer I at low concentration (84% at 0.01C*), reflecting selective stabilization under interfacial confinement. As concentration increases, competition between conformers I and III becomes more significant, leading to a redistribution of interfacial conformer populations with the fraction of conformer III increasing from 16% at 0.01C* to 90% at C*. This concentration-dependent reweighting of conformers at the interface closely parallels the experimentally observed shift in polymorph outcomes. We further tracked the time evolution of interfacial conformer populations over the course of the MD trajectories (Figure S12). The conformer populations exhibit only minor fluctuations throughout the analyzed MD trajectory, indicating that the concentration-dependent conformer bias is established during the early prenucleation stage and persists during local assembly. Thus, the interfacial mechanism operates as a coupled selection process: the gas–liquid interface biases conformer populations, while emerging FFA–FFA associations further stabilize the resulting local packing motifs.

To further examine the connection between conformer populations and polymorph outcomes, we compared the MD-derived conformer populations with the experimentally observed polymorph probabilities (Table S10). In bulk solution, the conformer-III-dominated population is consistent with the exclusive formation of form III under stirred bulk crystallization. At the gas–liquid interface, the shift from conformer-I enrichment (84%) at 0.01C* to conformer-III dominance (90%) at C* mirrors the experimental transition from form-I-rich outcomes to exclusive form III formation. Meanwhile, the competitive conformer population at 0.1C* corresponds to mixed polymorphic outcomes and the appearance of form IV.

In summary, the experimental and simulation results support a unified interfacial mechanism for polymorph selection. The gas–liquid interface induces molecular enrichment, orientational bias, and conformer redistribution, while also reordering the relative stability of conformer-dependent association motifs. These coupled effects reshape the prenucleation assembly landscape in a concentration-dependent manner, thereby biasing crystallization toward forms I, IV, or III.

## 3 Discussion

The combined experimental and simulation results indicate that polymorph selection in this system is governed not simply by bulk thermodynamic preference, but by a concentration-dependent kinetic selection mechanism operating at the gas–liquid interface. The interface provides a confined and anisotropic environment in which molecular enrichment, orientational restriction, and conformer-dependent association become coupled. Under these conditions, the balance among competing prenucleation assemblies is altered, allowing distinct structural motifs to be selectively favored as evaporation proceeds.

At low concentrations, the relatively dilute interfacial layer preserves orientational anisotropy. This orientation acts as a geometric preorganization factor that modulates the accessibility of specific association motifs at the interface. A surface-parallel arrangement confines FFA molecules into a more two-dimensional interfacial layer, facilitating lateral aromatic contacts and slipped π-stacked motifs associated with conformer I. This interpretation is consistent with the QM/MM results, showing that I–I hydrogen-bonded and π-stacked dimers are energetically preferred at the gas–liquid interface. These conformer-I-rich, surface-compatible motifs make form-I-related pathways more accessible at low concentration.

At intermediate concentration, increasing interfacial crowding introduces competing local arrangements, which provides a structural basis for the appearance of form IV and the coexistence of multiple polymorphic outcomes. As form IV accommodates multiple FFA conformations and diverse stacking geometries, it becomes accessible in this transition region, where form-I-like and form-III-like interfacial motifs coexist and compete. Given that form IV is a metastable polymorph, its appearance within this narrow concentration window may be postulated as a kinetically accessible outcome of the mixed interfacial assembly pathway.

At high concentrations, rapid interfacial enrichment and molecular crowding broaden the orientation distribution and weaken the surface-imposed orientational selectivity. Under these conditions, FFA–FFA interactions become increasingly dominant, allowing molecules to sample a wider range of three-dimensional association geometries and to amplify denser, more packing-efficient conformer-III-related hydrogen-bonded and π-stacked motifs. This shift is consistent with the energetic preference for III–III dimers in bulk-like environments and with the dominant formation of Form III at high concentrations.

This interpretation places the present system within a broader kinetic view of crystallization, in which phase selection depends not only on equilibrium stability but also on the accessibility, persistence, and amplification of precursor motifs under a given environment. In polymorphic systems, metastable forms may emerge when particular local organizations are preferentially formed or retained under nonequilibrium conditions, even if they are not the most stable bulk phase. From this perspective, the concentration-dependent polymorphism of FFA is a consequence of interfacial confinement prolonging specific molecular organizations that would otherwise relax or be averaged out in a bulk solution. The role of the interface is therefore to bias the prenucleation landscape toward different structural motifs as local concentration changes.

The present results are also consistent with previous observations that surfaces and interfaces can stabilize structural motifs that are absent or disfavored under bulk conditions. In this context, the gas–liquid interface should be regarded not merely as a passive boundary, but rather as a dynamic environment that reshapes molecular organization and biases crystallization pathways.^15,16^

An additional implication concerns static evaporation crystallization, which is often treated operationally as a bulk method. The present results suggest that this view is incomplete for polymorphic systems because static evaporation inherently maintains a persistent gas–liquid interface and evolving near-surface concentration gradients throughout nucleation. These conditions preserve the interfacial organization needed to bias early-stage assembly. This provides a structural explanation for the frequent stochasticity and limited reproducibility of polymorph outcomes in slow-evaporation crystal growth: relatively small changes in concentration history, surface stability, or interfacial perturbation may be sufficient to alter which assembly motif is preferentially amplified.

In summary, this work supports a general mechanism in which the gas–liquid interface reorders the relative accessibility of prenucleation assemblies through enrichment, anisotropic confinement, and conformer-selective association. Polymorph selection then emerges from the preferential amplification of different assembly pathways. This mechanism is expected to be most applicable to conformationally flexible organic molecules whose polymorphs differ in conformer-dependent hydrogen-bonding or π···π stacking motifs. For such molecules, competitive intermolecular interactions can stabilize different local association motifs, while anisotropic distributions of polar and aromatic/hydrophobic groups allow the gas–liquid interfacial solvation field to bias molecular orientation and conformer population. Evaporation-induced interfacial enrichment further provides a process-dependent condition under which these biased motifs can be amplified. This view is consistent with previous studies showing that gas–liquid interfaces can promote nucleation and, in some cases, select polymorphs distinct from those formed in the bulk, as reported for D-arabitol and posaconazole.^16,32^ Thus, this framework helps rationalize concentration-dependent polymorphism in FFA and may also be relevant to other evaporation-driven crystallization systems in which interfacial organization precedes nucleation.

## 4 Summary and Conclusions

In summary, we demonstrate that concentration-dependent polymorph selection in FFA during evaporation is governed by nucleation at the gas–liquid interface. Low, intermediate, and high concentrations lead predominantly to forms I, IV, and III, respectively, whereas disruption of the interface suppresses this selectivity and yields form III. *In situ* GIWAXS reveals distinct interfacial assembly pathways prior to nucleation, while molecular simulations show that the interface reshapes early-stage molecular organization through interfacial enrichment, orientational bias, conformer redistribution, and conformer-dependent association preferences. Together, these results establish the gas–liquid interface as an active structural selector in evaporation crystallization and provide a mechanistic basis for understanding and controlling polymorphism in molecular systems.

## 5 Materials and Methods

### 5.1 Materials

Flufenamic acid (>99%, form I) was obtained from Dalian Meilun Biotechnology Co., Ltd. Methanol was purchased from Aladdin Industrial Co., Ltd., of analytical grade. Methanol-d_4_ (TMA, NMR grade) was purchased from Shanghai TITAN Technology Co., Ltd. All of the chemicals were used without further purification.

### 5.2 Crystallization Experiment of FFA

#### 5.2.1 Surface Nucleation Experiment

FFA–methanol solutions with concentrations of 0.3445 g mL^–1^ (saturated, C*), 0.1723 (0.5C*), 0.0689 (0.2C*), 0.0345 (0.1C*), 0.0172 (0.05C*), 0.0069 (0.02C*), 0.0034 (0.01C*), and 0.0017 g mL^–1^ (0.005C*) were prepared by dissolving weighed FFA powder in methanol and filtered through syringe filter (0.22 μm) prior to use. For surface nucleation experiments, aliquots of the solutions were dropped onto a 20 cm × 20 cm silicon plate, forming thin liquid films. The samples were maintained at 25 °C under quiescent conditions to allow slow evaporation from the gas–liquid interface. Once nucleation occurred at the surface, the resulting crystalline material was collected immediately for phase identification.

Polymorphic forms were primarily identified by PXRD (Rigaku D/MAX 2500 X-ray diffractometer using Cu–Kα radiation, 2θ = 5–40°, scan rate 8° min^–1^), with reference patterns shown in Figure S1. When the amount of crystalline material was insufficient for PXRD analysis, micro-Raman spectroscopy (HORIBA JY) was employed as a complementary technique, using a 532 nm laser with a scan range of 50–3500 cm^–1^, with measured reference patterns shown in Figure S2. Each concentration was repeated 20 times to ensure statistical reliability.

#### 5.2.2 Stirred Bulk Nucleation Experiment

Bulk nucleation under perturbed conditions was examined by conducting crystallization experiments with continuous magnetic stirring. Filtered solutions of the same concentration series were transferred into 10 mL glass beakers (8 mL per beaker) and evaporated at 25 °C under stirring. Crystalline products were identified by PXRD, and crystal color was recorded as a supplementary indicator of polymorphic form (form I: white; form III: yellow; form IV: light yellow). Each concentration was repeated 10 times. Continuous stirring was used to suppress persistent gas–liquid interfacial organization.

#### 5.2.3 Validation Experiments (Static Evaporation and Surfactant Addition)

Static bulk crystallization experiments were performed using the same concentration series to allow simultaneous surface and bulk nucleation. After filtration, 8 mL of each solution was transferred into a 10 mL beaker and evaporated slowly at 25 °C without agitation. Crystals were collected upon appearance and characterized by PXRD. Each concentration was repeated 20–25 times.

To further probe the role of the gas–liquid interface, additional experiments were conducted by chemically perturbing the interface using a surfactant. Tween 80 (1 wt %) was added to the FFA–methanol solutions prepared as described above. After filtration, 8 mL of solution was placed in a 10 mL beaker and evaporated statically at 25 °C. The resulting crystalline solids were identified by PXRD. Each concentration was repeated five times. The addition of surfactant disrupts interfacial molecular organization without introducing bulk hydrodynamic mixing.

The evaporation rate measurements were performed under the same static evaporation conditions as those used for the crystallization experiments. FFA–methanol solutions of varying initial concentrations were placed in identical glass beakers, each with the same initial volume and exposed surface area. The solutions were maintained at 25 °C without agitation. The mass loss of each solution was recorded over time, and the evaporation rate was determined from the slope of the mass-loss profile during the early stage of evaporation.

### 5.3 Spectroscopy Analysis

#### 5.3.1 NMR Spectroscopy

^1^H NMR and 1D NOESY spectra were acquired on a 600 MHz Bruker Avance III spectrometer at 298 K. NMR samples were prepared by diluting FFA solutions of known concentrations with methanol-d_4_ containing TMS as an internal reference. Spectral processing and analysis were carried out by using MestReNova software.

#### 5.3.2 *In Situ* Grazing-Incidence Wide-Angle X-ray Scattering (GIWAXS)

*In situ* GIWAXS experiments were conducted at the BL02U2 beamline of the Shanghai Synchrotron Radiation Facility (SSRF) using X-rays with a wavelength of 0.6849 Å. FFA solutions were dropped onto a silicon plate mounted on the sample holder and allowed to evaporate under grazing-incidence conditions. Scattering patterns were continuously collected using a Pilatus 2 M detector positioned at a sample–detector distance of approximately 236 mm. The grazing-incidence angle was set to 0.15°, and the exposure time for each frame was 0.3 s. GIWAXS data were processed and analyzed using GIWAXS-Tools (Version 3).^33^

### 5.4 Molecular Modeling

#### 5.4.1 DFT Calculations for Conformational Analysis and NMR Chemical Shifts

The potential energy surface (PES) of FFA with respect to τ_1_ was calculated by optimizing molecular geometries with a step of 10° between −180° and 180° using the Gaussian 16 software package.^34^ The calculations were carried out using the SMD implicit solvation model^35^ for methanol at the B3LYP/6–311+G (d, p)^36^ level with a starting molecular geometry built from crystallographic data. ^1^H NMR chemical shifts of FFA were calculated using the Gauge-Independent Atomic Orbital method.^37^ The calculations were performed at the B3LYP/6–311+G (d, p) level.^36^

#### 5.4.2 Molecular Dynamics Model Construction

All explicit-solvent molecular dynamics (MD) systems were constructed using the Python-based version of ChemShell^31,38^ to generate solvated simulation boxes containing FFA and methanol. Three solution concentrations were considered by varying the number of FFA molecules within a fixed cubic simulation box, containing around 20,000 methanol molecules: 10 FFA (0.01C*), 100 FFA (0.1C*), and 1000 FFA (C*). Initial molecular coordinates were generated by random packing under excluded-volume constraints to eliminate steric overlaps. Force field assignment and topology generation were carried out using DL_FIELD 4.12 ^39^, and all MD simulations were performed with DL_POLY 5.3.0 ^40^ under explicit-solvent conditions. Methanol and FFA were modeled using all-atom OPLS-AA^41^ force field parameters. Methanol parameters were taken from the standard OPLS-AA set, while bonded terms for FFA were generated with the same force field scheme. Partial charges for FFA were derived by RESP fitting to the electrostatic potential obtained from gas-phase quantum-mechanical calculations at the ωB97X-D/def2-TZVP level of theory.^42^ Cross Lennard–Jones interactions were treated using standard combination rules. Long-range electrostatic interactions were described using the particle-mesh Ewald (PME) method,^43^ and van der Waals interactions were truncated at a finite real-space cutoff of 1 nm.

#### 5.4.3 Molecular Dynamics Simulations

All systems were first subjected to energy minimization to remove unfavorable contacts, followed by sequential equilibration in the canonical (NVT) and isothermal–isobaric (NPT) ensembles for 0.5 ns each. The equilibrated bulk simulation boxes had dimensions of approximately 10 × 10 × 10 nm^3^ for the three concentrations. Bulk simulations were subsequently propagated under NPT conditions to ensure proper sampling at the target temperature and pressure.

For gas–liquid interfacial simulations, an equilibrated bulk configuration at the corresponding concentration was converted into a slab geometry by extending the simulation box along the surface-normal direction, defined as the *z*-axis, from approximately 10 to 20 nm, while retaining periodic boundary conditions in all three dimensions. Vacuum regions of 5 nm thickness were introduced on both sides of the liquid slab, yielding two stable vapor–liquid interfaces and final slab boxes of approximately 10 × 10 × 20 nm^3^. The lateral box dimensions, *x* and *y*, were kept identical to those of the equilibrated bulk system to preserve the in-plane liquid density and avoid artificial lateral stress.

During production runs of the interfacial systems, simulations were carried out in the NVT ensemble with fixed cell dimensions to maintain a stable interface and prevent the collapse of the vacuum regions. Periodic boundary conditions were applied as appropriate for the bulk and slab geometries. MD trajectories were propagated using a 1 fs integration time step, and all covalent bonds involving hydrogen atoms were constrained using the LINCS algorithm.^44^

#### 5.4.4 Evaporation Simulations of the Prenucleation Stage

Evaporation crystallization at the gas–liquid interface was modeled using a quasi-evaporation protocol designed to reproduce the progressive increase in supersaturation during the early prenucleation stage of solvent evaporation without explicitly simulating macroscopic mass flux. Starting from an equilibrated slab configuration, methanol molecules were removed in discrete steps, followed by re-equilibration at each composition.

At the end of each evaporation cycle, 0.5% of the methanol molecules located in the region of the liquid slab that faces the vapor phase were deleted from the simulation system, thereby modeling the net loss of solvent from the liquid surface. After solvent removal, the system was re-equilibrated for 1 ns in the canonical ensemble to allow relaxation of the interfacial structure and solution composition before the next evaporation step. This remove–relax cycle was repeated iteratively 10 times, generating a controlled concentration trajectory that captures the key physicochemical features during the initial stage of the evaporation. Structural analyses were performed by randomly selecting 20 snapshots from the final 1 ns of each equilibrated trajectory, after the system had reached a steady state.

Temperature during the slab simulations was maintained using the velocity-rescale thermostat^45^ with a coupling constant of 1.0 ps. No pressure coupling was applied in order to preserve the vacuum regions and avoid artificial compression along the normal direction. Electrostatic interactions in the slab geometry were treated with particular care in GROMACS 2025.2 to reduce artifacts associated with the inhomogeneous dielectric environment along the *z*-direction.^46^

#### 5.4.5 Dimer Structure Energy Calculations

Single-point calculations of electrostatic embedding hybrid QM/MM models were carried out using ChemShell to evaluate the interaction energies of representative FFA dimers. Based on the classical MD trajectories, representative dimer configurations were extracted for subsequent QM/MM single-point calculations. This multiscale strategy allows efficient configurational sampling by classical MD, while using QM/MM to provide a higher-level description of conformer-dependent hydrogen bonding, π···π stacking, electrostatic, and dispersion interactions in the surrounding molecular environment. For both bulk and interfacial systems, 20 configurations were randomly selected from equilibrated production trajectories after steady state had been reached, and the reported quantities were obtained as averages over these configurations.

For each selected frame, a solvated cluster was constructed in which the QM region comprised approximately 120 atoms, including two interacting FFA molecules and their 10 nearest methanol molecules in the first solvent shell. The remaining surrounding methanol molecules (approximately 5000 atoms) were retained as the MM region to provide an explicit electrostatic embedding environment. A dual-basis strategy was employed within the QM region: the inner layer, centered on the FFA dimer, was treated with the def2-TZVP basis set, while the outer layer comprising the methanol molecules was treated with def2-SVP.^47,48^

Quantum-mechanical calculations using density functional theory were performed using the B97–2 ^49^ hybrid functional implemented in NWChem 7.0.0,^50,51^ while molecular mechanics interactions were evaluated using DL_POLY with the same OPLS-AA force field as used for the MD simulations. Binding energies were evaluated with the same embedding environment using the supramolecular scheme.

#### 5.4.6 Snapshots Analysis

Snapshot-based analyses were performed using GROMACS 2025.2 ^52^ and visualized through VMD.^53^ For each concentration and environment, 20 configurations were randomly selected from the final 1 ns of equilibrated trajectories. These snapshots were used to (i) assign conformation distributions (conformer I and conformer III) and (ii) quantify the orientation of FFA at the gas–liquid interface and in bulk solution.

Conformer assignment was based on the characteristic intramolecular torsion angle τ of FFA. The tilt angle θ, defined as the angle between the gas–liquid interface and the aromatic ring β plane, was used to analyze molecular orientations.

In addition to the final snapshot analysis, the time evolution of conformer populations was examined to clarify whether the conformational bias was established before or during interfacial assembly. For this analysis, snapshots were extracted every 1 ns from 1 to 10 ns of the production trajectory, and the fractions of conformers I and III were calculated as a function of simulation time.

## Supplementary Material



## Notes

Crystallographic data, physical property, original polymorphic outcomes data, standard PXRD and Raman Patterns, chemical structure of Tween 80, photographs of crystallization experiments, evaporation rate measurements, solubility data with and without Tween 80, *in situ* GIWAXS experimental setup, *in situ* GIWAXS maps at different initial concentrations, conformer-dependent packing motifs and intermolecular distance, representative motifs from MD trajectories, a comparison between distances derived from GIWAXS and crystal structures, representative MD snapshots, density profiles, and time evolution of conformer populations at the gas–liquid interface (PDF)
