# Supplementary material for: Interfacial Nucleation Pathways Governing Polymorph Selection Revealed by Synchrotron-Based In Situ GIWAXS and Molecular Simulations
Source: J Am Chem Soc. 2026 Jul 7;148(28):30224–37. doi: 10.1021/jacs.6c06437 (PMC13397546; doi:10.1021/jacs.6c06437)
Supplement: Supplementary file 1 [file ja6c06437_si_001.pdf]

Interfacial Nucleation Pathways Governing Polymorph Selection Revealed by Synchrotron-based In situ GIWAXS and Molecular Simulations

Yu Liu,[1,2,†] Xu Zhang,[1,3,†] XingFan Zhang,[3] Mingdong Zhou,[2] Xiaolong Li,[4] Fumin Wang,[1] Xubin Zhang,[1] Alexey A. Sokol,[3] You Lu,[6] Thomas W. Keal,[6] Weiwei Tang,[,1] C. Richard A. Catlow[*,3,5] and Junbo Gong[*,1]*

1. School of Chemical Engineering and Technology, Tianjin University, Tianjin 300072, People's Republic of China
2. School of Chemical Engineering, Shenyang University of Chemical Technology, Shenyang 110142, People's Republic of China
3. Kathleen Lonsdale Materials Chemistry, Department of Chemistry, University College London, London WC1H 0AJ, U.K.
4. Chinese Acad Sci, Shanghai Adv Res Inst, Shanghai Synchrotron Radiation Facility, Shanghai 201204, Peoples R China
5. School of Chemistry, Cardiff University, Park Place, Cardiff, CF10 3AT, UK
6. STFC Scientific Computing, Daresbury Laboratory, Warrington WA4 4AD, UK

Table of Contents

S1. Crystallographic, Structural, and Physical Property Data

Table S1. Crystallographic Data of FFA forms I, III and IV

Table S2. Torsion Angles of FFA in Different Conformers

Table S3. Physical Properties of FFA Forms I, III, and IV

S2. Crystallization Outcomes and Statistical Analysis

Table S4~S7. Original Polymorphic Outcomes Data

Figure S1. Standard PXRD Patterns of FFA Polymorphs

Figure S2. Standard Raman Patterns of FFA Polymorphs

Figure S3. Chemical structure of Tween 80

Figure S4. Photographs of Evaporation Crystallization Experiments

Figure S5. Evaporation-rate Measurements with Different Initial Concentrations

Table S8. Solubility of FFA in Methanol with and without Tween 80

S3. In-situ GIWAXS experiments

Figure S6. Experimental Set-up for In Situ GIWAXS Measurements

Figure S7. In-situ GIWAXS Maps at Different Initial Concentrations

Figure S8. Conformer-Dependent Packing Motifs and Intermolecular Distances

Figure S9. Representative Packing Motifs Extracted from MD Trajectories

Table S9. Comparison between Distances Derived from GIWAXS and Crystal Structures.

S4. MD results

Figure S10. Representative MD snapshots in Bulk Solution and at the Interface

Figure S11. Density Profiles along z-direction

Figure S12. Time Evolution of Conformer Populations at the Gas–liquid Interface

S1. Crystallographic, Structural, and Physical Property Data

Table S1 Crystallographic data of FFA form I、 form III and form IV

	Form I	Form III	Form IV
a (Å)	12.523 (4)	39.848 (2)	8.7589 (2)
b (Å)	7.868 (6)	5.107 (5)	11.6629 (3)
c (Å)	12.874 (3)	12.240 (7)	20.0229 (14)
α (°)	90	90	80.632 (6)
β (°)	95.2 (3)	92.47 (4)	81.041 (6)
γ (°)	90	90	73.532 (5)
V (A^{-3})	1263.27	1195.54	1922.33
Space group	$P2_1$/c	$C2$/c	$P1$(2)
Z	4	4	6
Z’	1	1	3
Density ($g.cm^{-3}$)	1.479	1.501	1.458
Packing coefficient	0.679	0.693	0.690
CCDC number	1160200	1160195	856659

Table S2 The torsion angles (τ_1 and τ_2) in different conformers of form I, form III and form IV

	Conformer I	Conformer III	Conformer IV-A	Conformer IV-B	Conformer IV-C
τ_1	-53.94	-176.48	-41.82	-156.12	-35.91
τ_2	179.21	-145.50	168.50	-165.05	158.31

Table S3 Physical properties of FFA Forms I, III, and IV[a]

		Form I	Form III	Form IV
Melting point/ ºC		134[1,2]	126[1,2]	122-124[2]
Stability at 25ºC		Metastable[1,3]	Stable[1,3]	Metastable[2,3]
Transition temperature/ ºC		42 (I ↔III)[1]	42 (I ↔III)[1]	~113 (IV to I)[2]
Solubility in methanol, x	313.15 K	/	0.0843[1]	/
	318.15 K	0.0906[1]	0.0928[1]	/
	333.15 K	0.1348[1]	0.1496[1]	Not reported

a. Data were compiled from literature reports.

S2. Crystallization Outcomes and Statistical Analysis

The resultant polymorph under different crystallization conditions were recorded in table S3-S6. Since the stochastic behavior, we calculated the polymorph probability under different crystallization conditions. The probability is calculated as follows:

$$P_i = \frac{N_i}{N_{total}}$$

Where P_i is the nucleation probability for form i, N_{total} represents the total number of experiments conducted at each condition and N_i represents the number of experiments obtaining form i. The polymorph probability reported here represents the frequency of obtaining each polymorphic form across repeated independent crystallization experiments under the same controlled experimental conditions. Note that it does not represent the relative amount or phase fraction of polymorphs within a single product. If two forms co-crystallized at one experiment, we recorded N_i as 0.5 for each form in the probability calculation.

The standard PXRD pattern used for form determination was given in figure S1 and the chemical structure of surfactant (Tween 80) was given in figure S2. The picture of the evaporation experiment at different concentrations was provided in figure S3.

Table S4 Original polymorphic results for surface nucleation with different initial concentrations

N	0.005C*	0.01C*	0.02C*	0.05C*	0.1c*	0.2C*	0.5C*	C*
1	I	I	I	IV	IV	III	III	III
2	I	I	I	IV	III	I	III	III
3	I	I	I	I	IV	III	III	III
4	I	I	IV	I	III	IV	III	III
5	I	I	I	III	I	IV	III	III
6	I	IV	I	IV	III	III	III	III
7	I	I	IV	IV	IV	III	III	III
8	I	I	IV	I	IV	III	III	III
9	I	IV	I	III	I	III	III	III
10	I	I	I	IV	IV	III	III	III
11	I	I	I	IV	IV	III	III	III
12	I	I	I	IV	III	III	III	III
13	I	I	IV	IV	III	IV	III	III
14	I	I	I	IV	IV	III	III	III
15	I	I	I	I	IV	IV	III	III
16	I	I	I	III	III	III	III	III
17	I	I	I	III	III	I	I+III	III
18	I	I	I	IV	IV	III	III	III
19	I	I	I	IV	III	III	III	III
20	I	I	I	IV	IV	III	III	III

Table S5 Original polymorphic results for static evaporation crystallization experiments with different initial concentrations.

N	0.005C*	0.01C*	0.02C*	0.05C*	0.1c*	0.2C*	0.5C*	C*
1	I	I	IV	I	I	III	III	III
2	I	I	IV	IV+I	I	III	III	III
3	I	IV	I	I	IV	IV	III	III
4	I	I	I	III	IV	I	III	III
5	I	I	III	I	III	I	III	III
6	I	I	I	IV	I	III	I	III
7	I	IV	III	I	IV	III	III	III
8	I	I	IV	III	IV	I	III	III
9	I	I	III	IV	I+IV	I	III	III
10	I	III	I	IV	I	III	III	III
11	I	III	I	I	I	III	I	III
12	I	I+III	I	I	I+III	III	I	III
13	I	I	IV+I	I+IV	III	III	III	III
14	I	IV	I	III	IV	IV	III	III
15	I	I	IV	I+III	III	III	III	III
16	I	I	I	I	I	III	III	III
17	I	IV	IV	IV	IV	III	I	III
18	I	IV	IV	I	IV	IV	III	III
19	I	I	I	IV	IV	III	III	III
20	I	I	I	III+I	I	III	III	III
21		I	IV					
22		I	III					
23		I	IV					
24		IV+I	I					
25		I	IV					

Table S6 Original polymorphic results for evaporation crystallization experiments under agitation with different initial concentrations.

N	0.005C*	0.01C*	0.02C*	0.05C*	0.1c*	0.2C*	0.5C*	C*
1	III	III	III	III	III	III	III	III
2	III	III	III	III	III	III	III	III
3	III	III	III	III	III	III	III	III
4	III	III	III	III	III	III	III	III
5	III	III	III	III	III	III	III	III
6	III	III	III	III	III	III	III	III
7	III	III	III	III	III	III	III	III
8	III	III	III	III	III	III	III	III
9	III	III	III	III	III	III	III	III
10	III	III	III	III	III	III	III	III

Table S7 Original polymorphic results for evaporation crystallization experiments with surfactant

N	0.005C*	0.01C*	0.02C*	0.05C*	0.1c*	0.2C*	0.5C*	C*
1	III	III	III	III	III	III	III	III
2	III	III	III	III	III	III	III	III
3	III	III	III	III	III	III	III	III
4	III	III	III	III	III	III	III	III
5	III	III	III	III	III	III	III	III

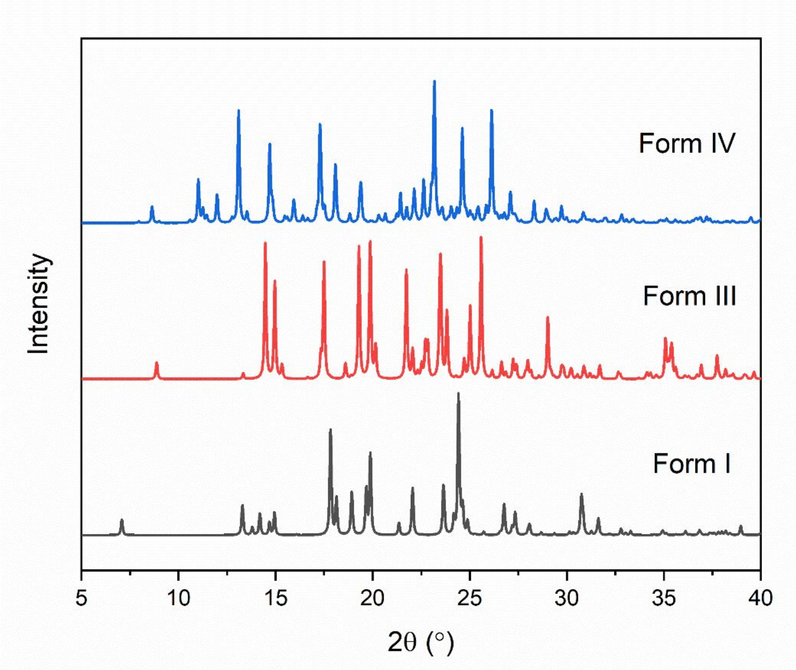

Figure S1 The standard PXRD patterns used for determining the polymorphic forms after crystallization experiments.

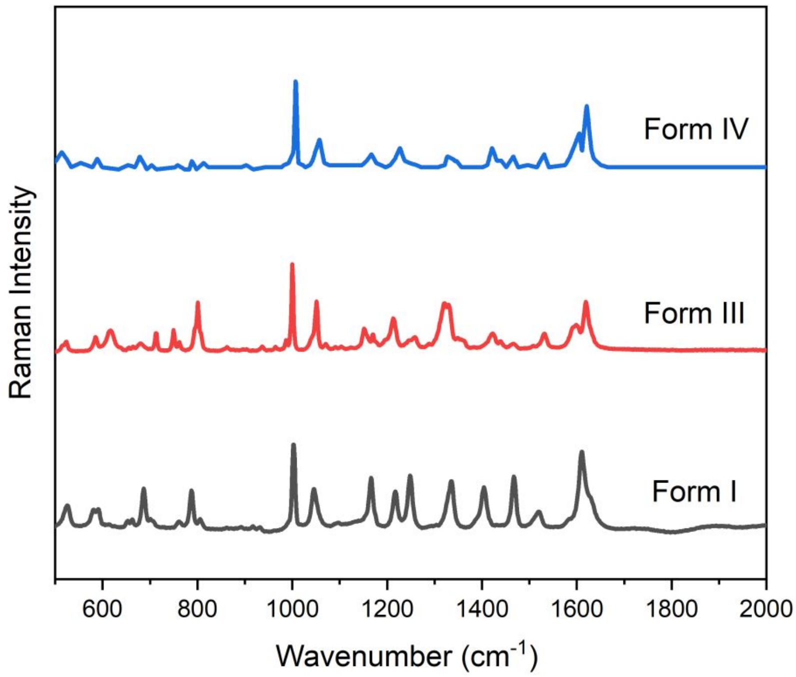

Figure S2. The reference Raman patterns used for polymorph assignment of the trace crystallization samples when PXRD analysis was not feasible.

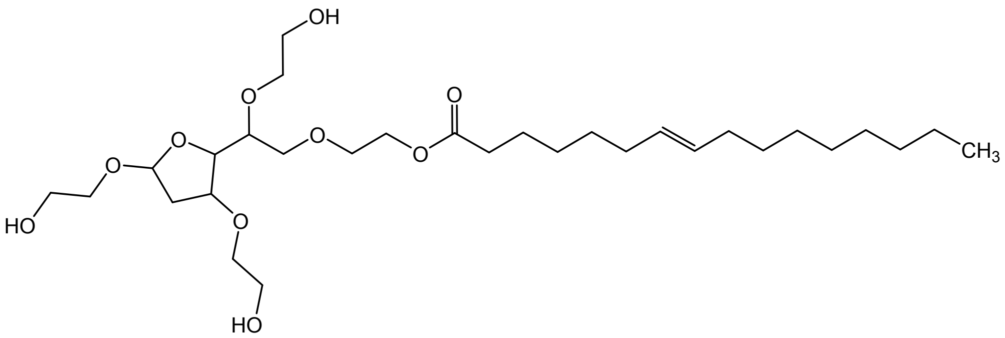

Figure S3 Chemical structure of Tween 80.

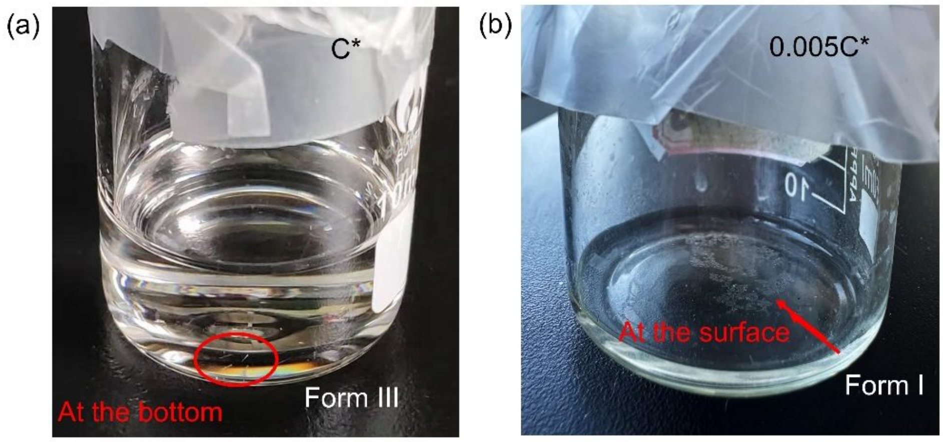

Figure S4 Photographs of evaporation crystallization experiments at C* and 0.005C*, yielding form III and form I, respectively, highlighting the distinct nucleation locations.

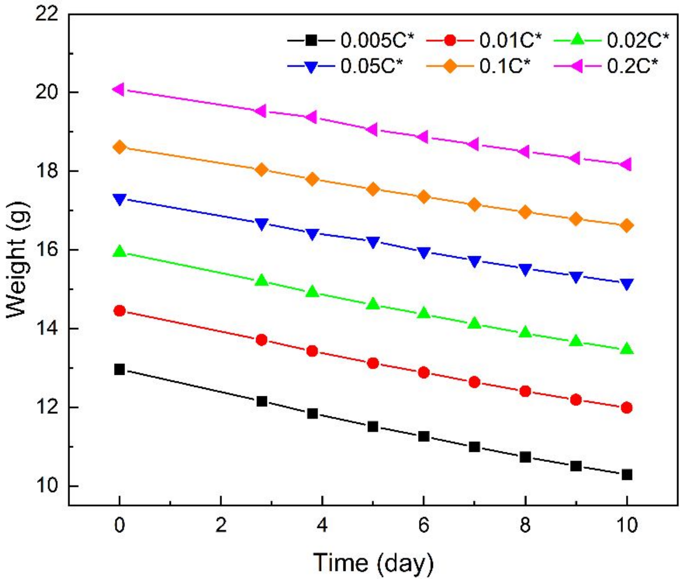

Figure S5. Figure S5. Evaporation-rate measurements of FFA–methanol solutions at different initial concentrations under static evaporation conditions at 25 °C. The mass-loss profiles show slow evaporation and comparable evaporation rates across studied concentrations.

Table S8 Mole-fraction solubility of FFA in methanol with and without Tween 80 at 298.15K.

Solvent system	FFA solubility
Methanol	0.04563
Methanol + Tween 80	0.04576

Solubility was measured under the same conditions used for the crystallization experiments. No additional solid phase was detected in the Tween-80-containing system. The measured solubility was 0.0496 in pure methanol and 0.0493 in the 1% Tween-80-containing methanol solution, indicating that Tween 80 had negligible influence on FFA solubility.

S3. In-situ GIWAXS experiments

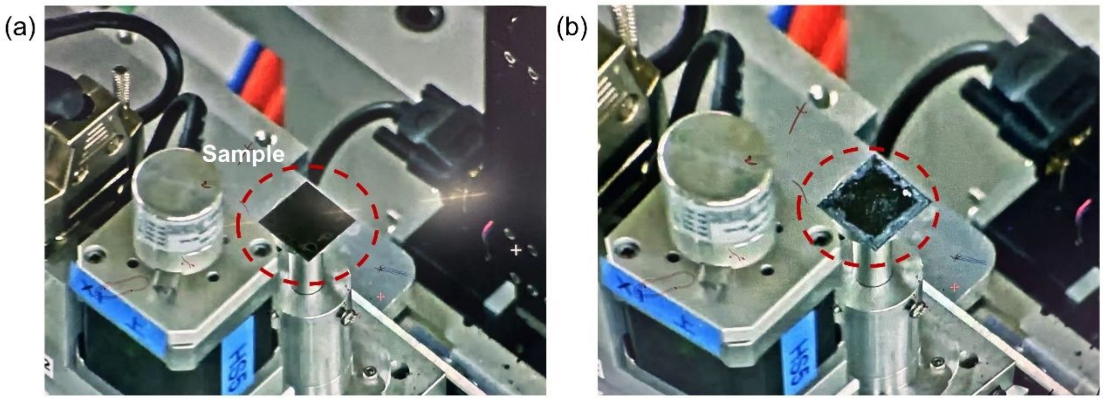

Figure S6 The visual windows for in-situ GIWAXS measurement used to record the crystallization time: (a) initial solution before evaporation and (b) sample after crystallization at 0.005C*.

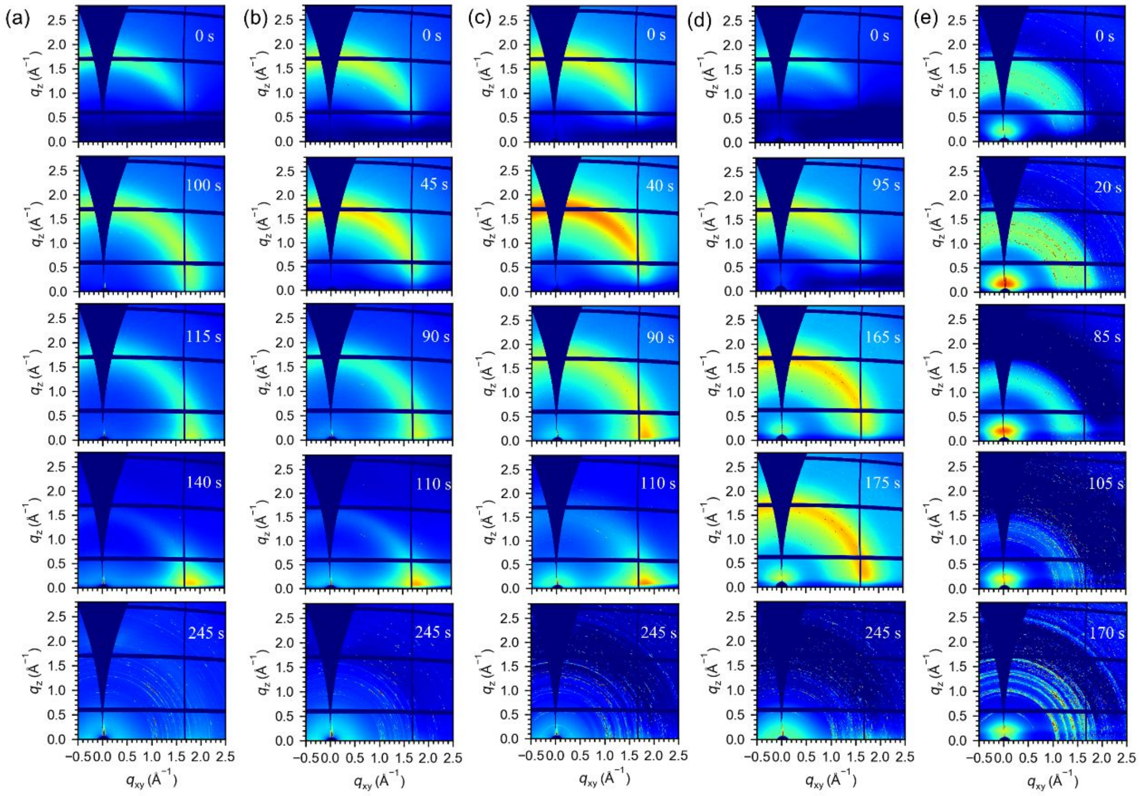

Figure S7 In-situ GIWAXS maps of FFA solutions at different initial concentrations: (a) 0.01C*, (b) 0.02C*, (c) 0.05C*, (d) 0.1C*, (e) C*.

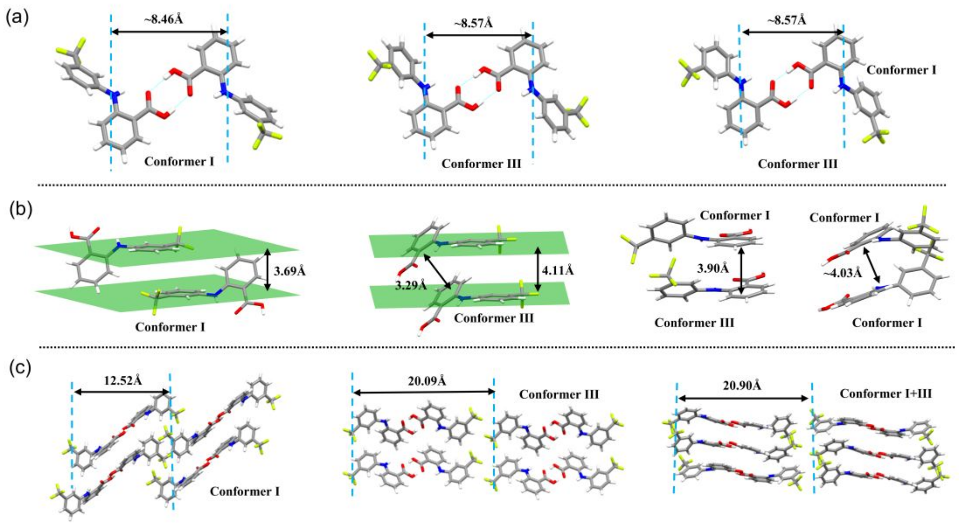

Figure S8. Schematic representation of conformer-dependent intermolecular distances and stacking motifs derived from crystal structure. (a) Hydrogen-bonded dimers. (b) π···π stacked dimers with distinct stacking distances and orientations. (c) Larger molecular clusters illustrating representative packing motifs associated with different conformer combinations.

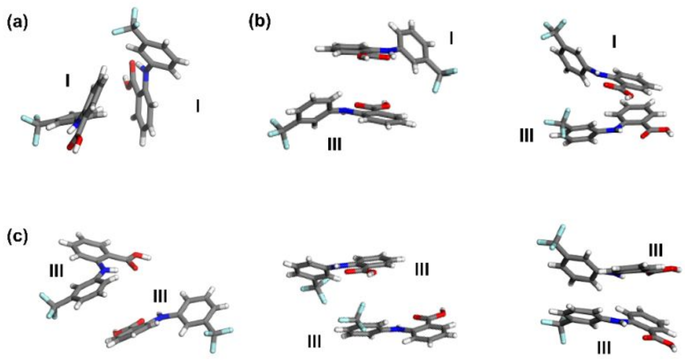

Figure S9. Representative local packing motifs extracted from MD trajectories in low concentration (a), medium concentration (b) and high concentration (c).

The representative local assemblies extracted from the MD trajectories exhibit conformer-dependent π-stacking and hydrogen-bonded association motifs resembling those observed in the crystal structures of Forms I, III, and IV. However, the molecular arrangements remain substantially more diffuse and less ordered than in the corresponding crystals. These observations suggest that the interfacial assemblies contain crystal-relevant local motifs but do not yet possess the long-range order

characteristic of crystalline nuclei.

Table S9. Comparison between GIWAXS-derived real-space distances and representative intermolecular distances from FFA crystal structures and MD-derived assemblies.

GIWAXS feature	q / Å^{-1}	d / Å	Suggested assignment[a]	Structural basis
Wide-angle diffuse arc	~1.7	~3.69	Short-range stacking correlation	Close to aromatic stacking distances in π-stacked motifs
Multiple wide-angle features	~1.2-1.6	~3.9-5.2	Offset stacking geometries	Consistent with conformer-dependent offset π-stacked arrangements
Wide arc (High Concentration)	~1.4	~4.48	Dominant local packing correlation	Consistent with conformer-dependent local arrangement
Low-q diffuse feature	~0.18-0.28	~22-35	Mesoscale density correlation	Attributed to inter-aggregate or cluster-level correlation

a. The GIWAXS-derived d-values represent characteristic real-space correlation lengths of diffuse interfacial scattering features and are not assigned to specific crystallographic reflections. The structural comparisons are based on representative intermolecular distances and packing motifs labelled in Figure S8.

S4. MD results

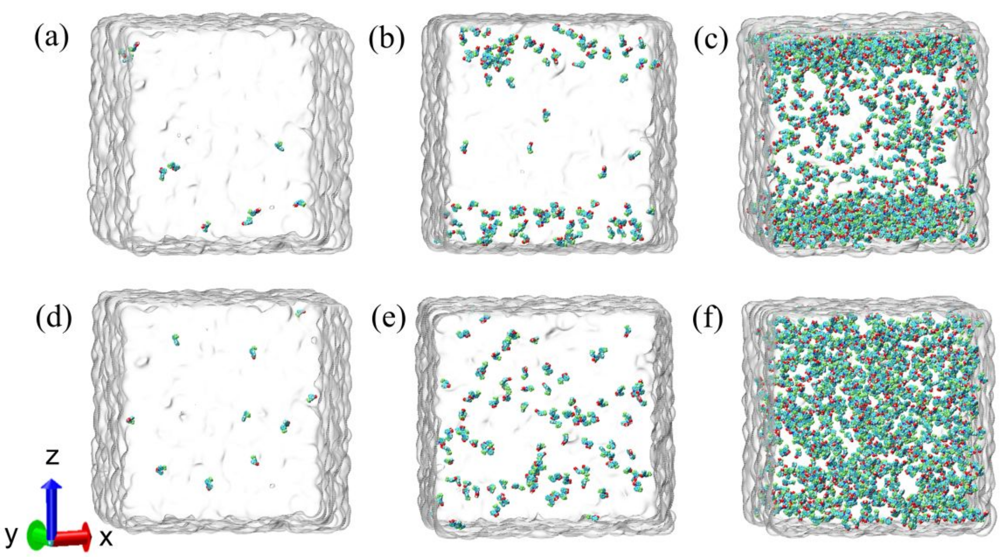

Figure 10. Representative MD snapshots illustrating the spatial distribution of FFA molecules at gas-liquid interface (a-c) and in bulk solutions (d-f) at different concentrations: (a, d) 0.01C*, (b, e) 0.1C*, (c, f) C*. Methanol molecules are rendered in white for clarity.

Table S10. Semi-quantitative comparison between MD-derived conformer populations and experimental polymorph outcomes.

Environment	Concentration	MD conformer I /%	MD conformer III /%	P (form I)/%	P (form IV)/%	P (Form III)/%	Dominant outcome
Bulk	0.01C*	33	67	0	0	100	Form III
Bulk	0.1C*	22	78	0	0	100	Form III
Bulk	C*	12	88	0	0	100	Form III
Interface	0.01C*	84	16	65	24	11	Form I
Interface	0.1C*	70	30	40	18	42	Competitive regime
Interface	C*	10	90	0	0	100	Form III

The density profiles were calculated as the number ratio $N_{\mathrm{FFA}}(z)/N_{\mathrm{MeOH}}(z)$within each bin along the z direction. The interfacial systems show pronounced increases in the local FFA/MeOH ratio near the gas–liquid interfaces, whereas the bulk systems remain relatively homogeneous. This analysis provides a semi-quantitative descriptor of effective interfacial enrichment rather than an absolute number-density or thermodynamic surface-excess measurement.

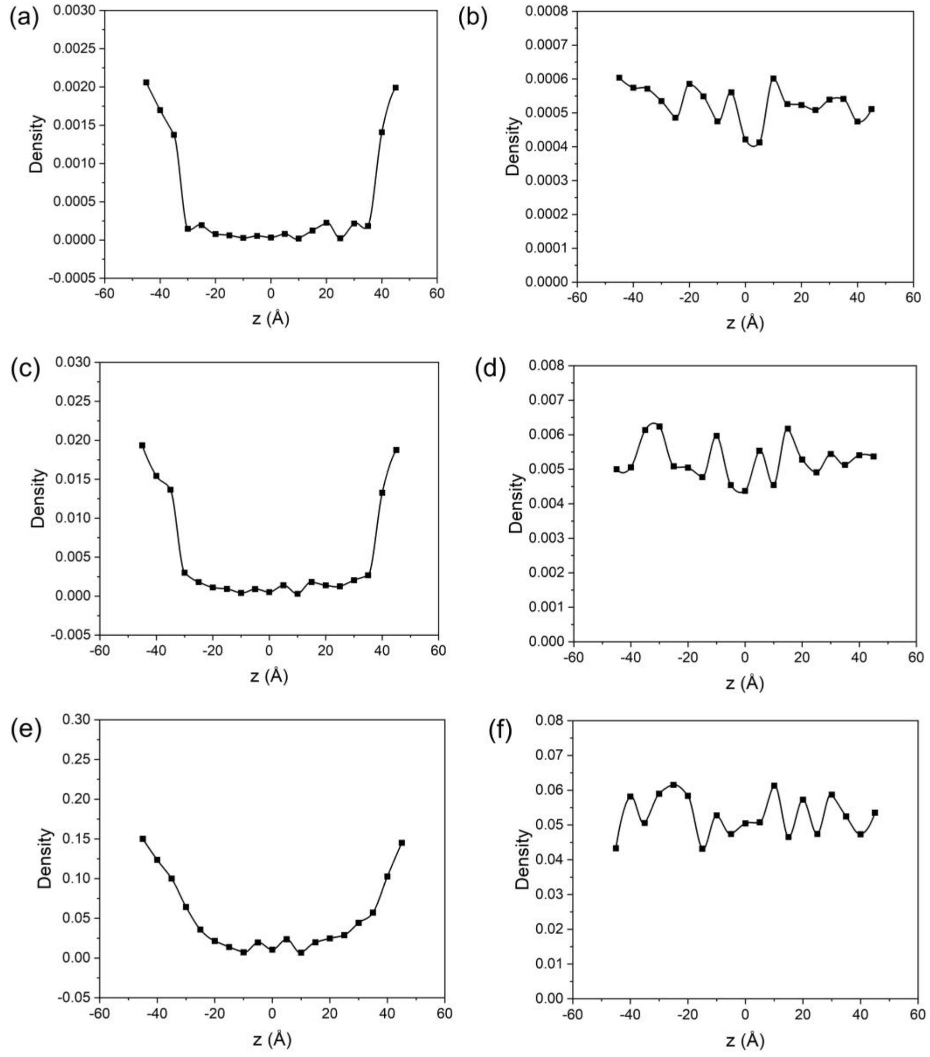

Figure S11. FFA number-density profile along z direction for gas–liquid interfacial and bulk simulation systems at different initial bulk concentrations. (a,b) 0.01C*; (c,d) 0.1C*; and (e,f) C*. Panels (a,c,e) show the gas–liquid interfacial systems, while panels (b,d,f) show the corresponding

bulk solution systems.

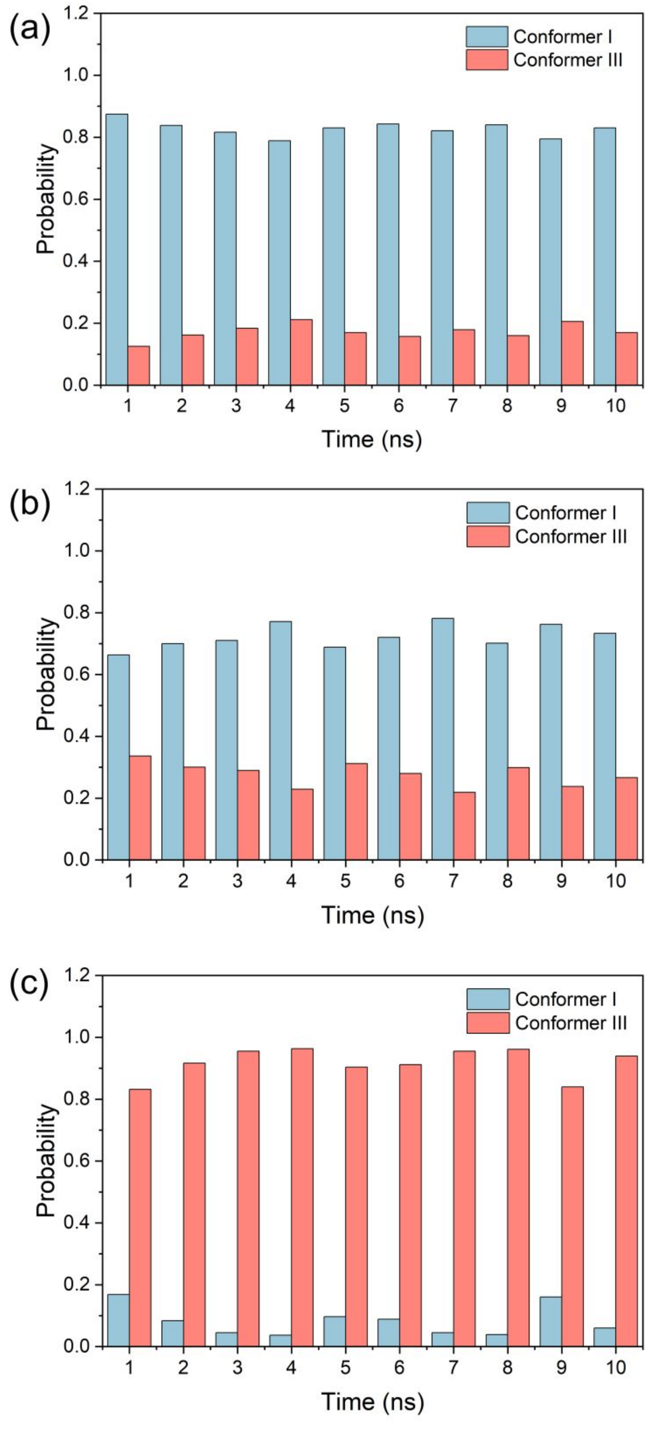

Figure S12. Time evolution of conformer populations at the gas–liquid interface under different initial concentrations. (a) 0.01C*, (b) 0.1C*, and (c) C*. The populations of conformer I and conformer III were calculated over consecutive 1 ns time windows from the MD trajectories.

References

(1) Marrero, V. R. V.; Berríos, C. P.; Rodríguez, L. D.; Stelzer, T.; López-Mejías, V. *Crystal Growth & Design* **2019**, *19*, 4101.

(2) Pang, Y. Y.; Buanz, A.; Gaisford, S.; Magdysyuk, O.; Williams, G. R. *Molecular Pharmaceutics* **2022**, *19*, 1477.

(3) López-Mejías, V.; Kampf, J. W.; Matzger, A. J. *Journal of the American Chemical Society* **2012**, *134*, 9872.
